# Worldwide Distribution and Extracutaneous Manifestations of Henoch-Schönlein Purpura in Adults: Narrative Review

**DOI:** 10.2196/49746

**Published:** 2024-01-25

**Authors:** Blair W Harris, Luke Maxfield, Abigail Hunter, Mandy Alhajj, Byung Ban, Kayd J Pulsipher

**Affiliations:** 1 Department of Dermatology Sampson Regional Medical Center Campbell University Clinton, NC United States; 2 Department of Dermatology Hospitals Cleveland Medical Center Case Western Reserve University Cleveland, OH United States; 3 Department of Rheumatology MedStar Georgetown University Hospital Washington, DC United States

**Keywords:** extracutaneous manifestations, HSP, Henoch-Schönlein purpura, immunoglobulin A vasculitis, IgAV, IgA vasculitis, narrative review

## Abstract

**Background:**

Henoch-Schönlein purpura (HSP), a leukocytoclastic small vessel vasculitis, exhibits both cutaneous and systemic manifestations. While predominantly observed in childhood, it may manifest in adults with more pronounced systemic involvement. Furthermore, HSP is a global phenomenon showcasing epidemiological and systemic variances.

**Objective:**

This study aims to scrutinize extracutaneous manifestations in adults with HSP, discerning distinctions according to geographical regions on a worldwide scale.

**Methods:**

A comprehensive search encompassing PubMed, Embase, Cochrane Library, and Web of Science was executed, covering papers published from January 1, 1970, to December 1, 2019. Keywords used included “Henoch-Schönlein purpura,” “henoch schonlein purpura+adult,” “IgA vasculitis+adult,” “HSP+adult,” and “IgAV.” A total of 995 publications were identified, from which 42 studies encompassing 4064 patients were selected, with a predominant focus on cases reported in Asia, Europe, and the Americas.

**Results:**

Among adults afflicted with HSP, European patients exhibited a higher propensity for male predominance (*P*<.001), gastrointestinal involvement (*P*<.001), and musculoskeletal complications (*P*<.001). Conversely, patients from the Americas were least likely to experience genitourinary involvement (*P*<.001).

**Conclusions:**

HSP demonstrates a variance in distribution and extracutaneous manifestations within distinct geographical boundaries. In the adult population, European patients exhibited a higher prevalence of male gender and gastrointestinal and musculoskeletal involvement. Asian patients were more predisposed to genitourinary involvement when compared to their American counterparts. The establishment of prospective studies using standardized reporting measures is imperative to validate the relationships unveiled in this investigation.

## Introduction

Henoch-Schönlein purpura (HSP), also known as immunoglobulin A vasculitis (IgAV), stands as the most prevalent form of vasculitis in childhood [1]. This condition exhibits a considerable disparity in incidence between children and adults, with palpable purpura constituting a hallmark feature in both populations [1-7]. Approximately 90% (135/150) of HSP cases manifest within the first decade of life. Notably, the United States reports an annual incidence in children ranging from 6.1 to 20.4 cases per 100,000. In the United Kingdom and France, children aged 17 years or younger demonstrate an annual incidence of approximately 20 to 70 cases per 100,000. It is noteworthy that Asian studies have documented an even higher incidence, reaching 56 cases per 100,000 [2,3,7-14].

In contrast, the annual incidence of HSP in adults exhibits a lower prevalence, estimated to range from 1.4 to 5.1 cases per 100,000, with a heightened frequency observed during the fifth and sixth decades of life [8-11]. In both pediatric and adult populations, HSP has been reported to display a male predilection, barring exceptions identified in 2 Korean studies [11-13].

Despite individual investigations focusing on the correlation between cutaneous manifestations and systemic involvement, no prior studies have undertaken a comprehensive assessment of the global geographical disparities concerning extracutaneous manifestations in adult patients. Our primary aim, therefore, is to meticulously scrutinize the existence and distribution of extracutaneous manifestations in adult patients with HSP, stratified by geographic regions across the world.

## Methods

### Search Parameters

To comprehensively assess extracutaneous manifestations in adults with HSP, an exhaustive review of the literature was conducted. This review encompassed the databases PubMed, Embase, Cochrane Library, and Web of Science, scrutinizing papers published from January 1, 1970, through December 1, 2019. A search was facilitated using the following keywords: “Henoch-Schönlein purpura” OR “henoch schonlein purpura+adult,” “IgA vasculitis+adult,” “HSP+adult,” and “IgAV.” A total of 995 publications were initially identified.

### Inclusion Criteria

Stringent inclusion criteria were applied, focusing exclusively on papers presenting original data that contained pertinent information regarding gastrointestinal (GI), genitourinary (GU), and musculoskeletal (MSK) involvement. Specifically, GI involvement was delineated as the onset of abdominal pain, melena, or hematochezia; MSK involvement was defined by the emergence of new arthritis or arthralgia; and GU involvement was ascribed to the appearance of new proteinuria, hematuria, acute kidney injury, or the exacerbation of chronic kidney disease. Individual case reports and publications limited solely to pediatric patients were excluded from the analysis.

### Screening Process

This meticulous screening process resulted in the inclusion of 42 eligible studies that adhered to the predefined criteria. The majority of the reported cases were drawn from 3 predominant regions, namely, Asia, Europe, and the Americas. Subsequently, patients were categorized according to their respective geographical regions, and a comparative analysis was performed to discern the number of cases and the mean involvement of the GI, GU, and MSK systems within each region. Statistical analyses, including omnibus tests and post hoc pairwise comparisons, were executed using MedCalc (version 19.1; MedCalc Software Ltd).

## Results

### Age and Gender by Geography

A total of 42 studies incorporating data from 4064 adult patients were included in this comprehensive analysis. These studies were divided into 23 European studies, 17 Asian studies, and 4 papers published in North and South America. Notably, the age of onset in Asian patients was significantly earlier, with an average of 29.8 (SD 7.02) years, compared to their European counterparts (mean 49.3, SD 9.14 years; *P*<.001) and individuals in the Americas (mean 48.6, SD 4.17 years; *P*<.001). In terms of gender distribution, a marked discrepancy emerged, with male patients exhibiting a higher prevalence in Europe (n=997, 62.2%), while both genders demonstrated a relatively equitable distribution in Asia and North and South America (n=135, 47.7% vs n=1120, 51.4%; *P*=.12).

### Extracutaneous Manifestations by Geography

Furthermore, the clinical presentation of HSP exhibited noteworthy regional variations. Europeans displayed a higher propensity for GI involvement, affecting 58.2% (n=932) of patients, a percentage significantly greater than the 31.4% (n=89) observed in the Americas (*P*<.001) and the 44.7% (n=974) in Asian populations (*P*<.001). Additionally, MSK involvement was notably prevalent among Europeans, with 57.9% (n=928) of individuals manifesting such symptoms. This proportion exceeded the figures observed in the Americas (n=135, 47.7%; *P*<.001) and Asia (n=1034, 47.4%; *P*<.001). Interestingly, among adults in Asia with HSP, GU involvement was the most frequent, impacting 72.3% (n=1575) of patients, although this did not display a statistically significant difference from the 67.2% (n=1077) observed in European populations (*P*=.08). Conversely, individuals in North and South America exhibited the lowest likelihood of GU involvement at 47% (n=133; *P*<.001). A comprehensive summary of demographics and the extent of extracutaneous organ involvement can be found in Table 1.

**Table 1:** Age, gender, and extracutaneous involvement of Henoch-Schönlein purpura separated by geographic region (N=4064).

Region	Total patients, n (%)	Age (years), mean (SD)	Male patients, n (%)	Female patients, n (%)	Gastrointestinal involvement, n (%)	Musculoskeletal involvement, n (%)	Genitourinary involvement, n (%)
Europe	1602 (39.4)	49.3 (9.14)	997 (62.2)	605 (37.8)	932 (58.2)	928 (57.9)	1077 (67.2)
Americas	283 (6.9)	48.6 (4.17)	135 (47.7)	148 (52.3)	89 (31.4)	135 (47.7)	133 (47)
Asia	2179 (53.6)	29.8 (7.02)	1120 (51.4)	1059 (48.6)	974 (44.7)	1032 (47.4)	1575 (72.3)

## Discussion

### Pathophysiology

The etiology of HSP remains elusive; however, this systemic vasculitis is widely regarded as an immune-mediated disorder, characterized by the deposition of immunoglobulin A (IgA) complexes, which underlie the pathological alterations observed in the skin, kidneys, GI tract, and joints [15,16]. Various triggers have been postulated for the onset of HSP, encompassing recent upper respiratory infections, medications, and malignancies [13,17]. Notably, HSP appears to exhibit a seasonal predilection with a peak incidence during the winter months, while occurrences during the summer months are relatively rare [8,17].

### Clinical Manifestations

HSP in the adult population is frequently associated with heightened disease severity and less favorable outcomes, particularly when it involves the integumentary system, renal function, and systemic vasculitic manifestations, in stark contrast to its typically benign and self-limiting course in children [11,16,18-20]. On rare occasions, HSP may extend its impact to include the pulmonary, cardiac, or nervous systems. The hallmark clinical features of HSP encompass the characteristic purpuric rash, joint pain, abdominal discomfort, edema, and hematuria [4,21,22].

Cutaneous manifestations of HSP commence as erythematous macules or urticarial papules, evolving into nonblanching palpable purpura, which symmetrically affect extensor surfaces, notably the buttocks and lower extremities. In some cases, involvement may extend to the trunk, face, and upper extremities [2]. Hemorrhagic bullae and vesicles appear to be more prevalent in older individuals [1,22].

There exists a divergence of evidence regarding the correlation between the severity of skin lesions and the extent of renal involvement and overall disease trajectory. Some investigations suggest that renal involvement is more frequent in individuals displaying skin direct immunofluorescence (DIF), indicative of immunoglobulin M (IgM) deposition and necrotic bullous skin lesions [1,23]. Conversely, other studies have failed to establish cutaneous IgM as a reliable indicator of renal or systemic disease in adult patients with HSP [24]. It is noteworthy that younger males presenting with generalized purpura and concurrent bowel involvement tend to experience less favorable outcomes, thus implying that the extent of skin involvement may serve as a predictive factor for the disease course and potentially guide therapeutic decisions [25].

Joint pain stands as a prevalent clinical manifestation of HSP, with its occurrence noted in over 60% of adult cases, with a higher likelihood observed in those 60 years and younger of age [26,27]. Joint disease may manifest in the form of arthritis or arthralgias, typically exhibiting a symmetric distribution, and most frequently impacting the knee and ankle joints [27]. Importantly, joint involvement typically resolves without enduring sequelae [28].

GI involvement, in conjunction with renal complications, constitutes a significant source of morbidity in adult patients with HSP [26]. Roughly two-thirds of HSP presentations include GI manifestations, most commonly manifesting as abdominal pain. Predominant abdominal symptoms encompass vomiting, diarrhea, periumbilical pain, and hematochezia. Notably, intussusception occurs in approximately 5% of patients, representing a significant GI complication. Other less frequent complications encompass bowel ischemia or infarction, necrosis, perforation, stricture formation, and GI hemorrhage [2,21,22,29].

Renal involvement is a common occurrence in HSP, yet its severity displays considerable variability. Indications of renal compromise manifest as hematuria and soft tissue edema due to proteinuria. Hematuria associated with HSP is typically macroscopic and may coincide with relapses of purpura or occur long after the resolution of extrarenal manifestations. The extent of proteinuria and the development of nephrotic syndrome exhibit a variable course, potentially leading to deterioration in glomerular filtration rate, azotemia, or end-stage renal failure. Predictors of renal involvement encompass recent infectious history, pyrexia, extension of purpura to the trunk, and biological markers of inflammation [22,23,30-32].

Notable predictors of adverse outcomes comprise renal insufficiency, hypertension, and the parameter of “young age” in adult patients [33-35]. Age at the onset of HSP has been postulated as a pivotal factor influencing disease severity and prognosis. Studies conducted by Hung et al [36] identified patients aged 20 years and older, male gender, bloody stools, and a rash persisting beyond 1 month as adverse prognostic factors for HSP. Schaier et al [37] reported that older patients with HSP presenting with renal involvement exhibited poorer outcomes than those aged 60 years and younger.

HSP nephritis stands as the most serious complication of HSP, with an incidence ranging from 20% to 80%. An adverse prognosis is particularly pronounced in patients presenting with nephrotic syndrome, renal failure, and, notably, hypertension at the time of diagnosis [38]. The presence of HSP nephritis aligns with the severity of renal histopathological changes [30-32,34].

### Diagnosis

The diagnosis of HSP fundamentally relies upon clinical manifestations. In adults, biopsy is more frequently used to confirm the diagnosis, while pediatric patients typically necessitate biopsy only in cases of atypical presentations. While no specific diagnostic tests for HSP exist, a normal platelet count and coagulation studies play a crucial role in excluding other diseases that may be present with palpable purpura [8,17].

The diagnostic criteria for HSP, developed by European League Against Rheumatism/Paediatric Rheumatology International Trials Organisation/Paediatric Rheumatology European Society, exhibit a sensitivity of 100% and a specificity of 87%. The diagnostic criterion mandates the presence of purpura or petechiae, characterized by a lower limb predominance, along with a minimum of one of four of the following criteria [39]: (1) acute onset of diffuse abdominal pain, (2) histopathological evidence demonstrating leukocytoclastic vasculitis or proliferative glomerulonephritis with predominant IgA deposits, (3) acute onset of arthritis or arthralgia, and (4) renal involvement, as indicated by proteinuria or hematuria.

For diagnosing the cutaneous vasculitis associated with HSP, the gold standard is a skin biopsy illustrating leukocytoclastic vasculitis in postcapillary venules, with the presence of IgA deposition, with or without eosinophils [22,40]. Notably, individuals aged 40 years and older, lacking eosinophils on skin biopsy, are reported to exhibit nearly a 3-fold heightened risk of developing renal involvement compared to those with eosinophils observed on skin biopsy [22,40].

HSP lacks specific biomarkers for diagnosis; nevertheless, certain markers hold effectiveness in monitoring disease activity and prognosis. DIF may reveal perivascular IgA and C3 deposition; however, individuals who otherwise meet clinical HSP criteria may not display IgA deposition on DIF [22]. In cases where diagnostic uncertainty exists or severe renal involvement is evident, a renal biopsy may be deemed necessary. Renal biopsies may illustrate mesangial hypercellularity (grades I through VI) and crescents on light microscopy. Characteristic of HSP nephritis is the presence of granular mesangial IgA and C3 deposition on light microscopy (with IgM and immunoglobulin G to a lesser extent) [13,22]. It is noteworthy that, on renal biopsy, the pathognomonic granular IgA and C3 deposition in the mesangium is indistinguishable from IgA nephropathy [22]. Moreover, the extent of interstitial fibrosis, the percentage of sclerotic glomeruli, and the presence of glomeruli displaying fibrinoid necrosis on renal biopsy have been associated with an unfavorable renal prognosis [27].

### Treatment

The management of adult IgAV has garnered limited investigation and remains a subject of controversy [26,41]. Notably, adults often necessitate more aggressive therapeutic approaches compared to pediatric patients. The mainstay of treatment involves supportive care and corticosteroids, complemented by varying use of immunosuppressive agents and plasma exchange [42].

Corticosteroids contribute to the swift resolution of renal manifestations and serve as a valuable tool in the management of joint and abdominal pain along with the duration of skin lesions. However, their efficacy in preventing palpable purpura or complications such as glomerulonephritis, bowel infarction, or intussusception remains unproven [11,26,41-43].

Immunosuppressive agents, including cyclophosphamide, cyclosporine, and rituximab, have been subjects of study in the context of HSP treatment. In instances marked by severe organ involvement and life-threatening complications, corticosteroids and immunosuppressive drugs are often initiated. Nevertheless, the augmentation of immunosuppressant agents to corticosteroid regimens does not appear to confer additional benefits when juxtaposed with the use of corticosteroids in isolation. Pillebout et al [27], for instance, conducted a comparative analysis between corticosteroids alone and corticosteroids combined with cyclophosphamide in patients with biopsy-confirmed IgAV and discerned no discrepancy at 12 months with regard to remission rates, renal outcomes, and adverse events. However, it is noteworthy that overall survival was more favorable in the corticosteroids plus cyclophosphamide group [26,27,41]. In a study by Maritati et al [44], rituximab, a B-cell depleting antibody, exhibited safety and efficacy in the treatment of adult-onset IgAV, with 20 of 22 patients achieving remission, although 7 of those 20 experienced disease relapse [44].

An illustrative case series by Augusto et al [45] highlighted the potential benefits of combining corticosteroids and plasma exchange in the treatment of severe HSP in adults. This approach yielded swift improvements in the patient Birmingham Vasculitis Activity Score, estimated glomerular filtration rate, and proteinuria, culminating in positive long-term outcomes at 6 and 12 months [45]. Nevertheless, renal involvement can precipitate end-stage renal failure, and it may manifest rapidly, necessitating the imperative need for dialysis or renal transplant, notwithstanding the concerns surrounding disease relapse [41]. Encouragingly, in 1 case series, none of the 12 transplant recipients lost their grafts due to relapse [41]. However, it should be acknowledged that renal transplant recipients have been subject to relapses, with 1 instance suggesting a potential role for plasmapheresis in addressing disease recurrence [46].

### Limitations

The primary limitation of this review is related to the simplicity of our search strategy. The volume of publications indexed in PubMed, Embase, Cochrane Library, and Web of Science in combination with the stringent screening process used limited our review to the 42 papers included in the results.

### Conclusions

Our comprehensive review underscores the noteworthy observation that adults afflicted with HSP frequently manifest pronounced extracutaneous involvement, with a proclivity toward progressive renal disease. Furthermore, it highlights the prospect of regional disparities in the risk of developing extracutaneous manifestations associated with HSP. To corroborate the relationships elucidated in this investigation, there is a compelling need for prospective studies that use standardized reporting measures.

## Abbreviations

DIF: direct immunofluorescence
GI: gastrointestinal
GU: genitourinary
HSP: Henoch-Schönlein purpura
IgA: immunoglobulin A
IgAV: Immunoglobulin A vasculitis
IgM: immunoglobulin M
MSK: musculoskeletal
